# Combination of Laparoscopic Sutureless Gastropexy and Ovariectomy in Dogs

**DOI:** 10.3390/ani15152205

**Published:** 2025-07-27

**Authors:** Marta Guadalupi, Roberta Belvito, Alberto Maria Crovace, Pasquale Mininni, Francesco Staffieri, Luca Lacitignola

**Affiliations:** 1 Sez. Cliniche Veterinarie e p.a., Dipartimento DiMePRe-J, Campus di Medicina Veterinaria, Università Degli Studi di Bari “Aldo Moro”, s.p. per Casamassima km 3, Valenzano, 70010 Bari, Italy; marta.guadalupi@uniba.it (M.G.); r.belvito@phd.uniba.it (R.B.); francesco.staffieri@uniba.it (F.S.); 2 Dipartimento di Medicina Veterinaria, Università degli Studi di Sassari, 07100 Sassari, Italy; acrovace@uniss.it

**Keywords:** laparoscopy, dog, gastropexy, ovariectomy, GDV

## Abstract

Prophylactic gastropexy is increasingly recommended in large-breed dogs predisposed to gastric dilatation-volvulus (GDV), particularly when combined with other elective procedures such as ovariectomy to reduce surgical trauma and anesthesia exposure. This prospective clinical study aimed to evaluate the feasibility, safety, and outcomes of a combined laparoscopic ovariectomy (LOVE) and total laparoscopic gastropexy with absorbable fixation straps (TLG-SS) using a standardized three-port minimally invasive approach. Six female dogs of GDV-prone breeds underwent the combined procedure. Surgical times, intraoperative and postoperative complications, and follow-up outcomes were recorded. The mean total operative time was 29.0 ± 3.52 min, with ovariectomy and gastropexy requiring 7.5 ± 1.38 and 9.33 ± 2.58 min, respectively. No major intraoperative complications occurred, and no conversion to open surgery was necessary. Postoperative recovery was uneventful in all cases, with only one minor portal site reaction observed. Owner satisfaction was excellent. The use of absorbable fixation straps simplified the gastropexy procedure and reduced operative time compared to other laparoscopic techniques. These findings suggest that the combined LOVE and TLG-SS procedure is technically feasible and well-tolerated in a small cohort of large-breed dogs, supporting its potential integration into clinical protocols pending further validation.

## Simple Summary

Large dog breeds are at risk of developing a life-threatening condition called gastric dilatation-volvulus (GDV), where the stomach fills with gas and twists. To prevent this, veterinarians can perform a surgery called gastropexy, which secures the stomach to the abdominal wall. This study tested a modern, less invasive version of the surgery that avoids traditional stitches and uses small absorbable straps instead. Six healthy female dogs of breeds prone to GDV were selected. The researchers combined two surgeries—removal of the ovaries and stomach fixation—into one minimally invasive procedure using three small incisions. The surgeries were completed quickly, with minimal discomfort, and no major complications occurred. All dogs recovered well, needed no extra pain relief, and were sent home a few hours after surgery. This combined approach may offer a safer, simpler, and faster way to prevent GDV and spay female dogs, reducing the need for multiple surgeries and anesthesia sessions. This case series suggests that the combined technique is technically feasible and well-tolerated, and may represent a practical approach to managing at-risk dogs while streamlining surgical workflow for veterinary teams.

## 1. Introduction

In recent years, prophylactic gastropexy has gained popularity as an elective procedure in dogs considered at risk for gastric dilatation-volvulus (GDV), even in the absence of acute symptoms [1]. Although numerous techniques have been described for performing gastropexy, no universal ‘gold standard’ has been established. However, minimally invasive techniques are increasingly adopted, particularly in elective settings, because are associated with reduced postoperative pain, quicker recovery, and decreased tissue trauma [2,3,4,5,6].

Among the various techniques described for gastropexy, traditional open or laparoscopic-assisted approaches often rely on incisional pexy, performed by creating an incision through the seromuscular layer of the stomach and the abdominal wall, followed by suturing the two surfaces together using monofilament absorbable sutures. Recent advancements in minimally invasive surgery have introduced simplified laparoscopic approaches that maintain comparable long-term efficacy while reducing surgical complexity and operative time.

In particular, techniques involving thermal scarification of the seromuscular gastric and peritoneal layers, followed by the application of a single suture line—either continuous or interrupted—using barbed monofilament materials (such as polydioxanone or glycomer 631), have gained attention for their procedural efficiency. Multiple studies have demonstrated that this combination (thermal incision plus barbed suture) offers secure and long-lasting adhesion of the stomach to the abdominal wall, with significantly reduced surgical time compared to conventional intracorporeal knot tying, and without compromising the stability of the pexy over time [5,7,8,9,10,11].

These sutureless or semi-sutureless techniques are often favored for their ease of execution, lower technical demands, and reproducibility, especially in settings where laparoscopic skills may be limited or still in development [5,9,11]. Furthermore, barbed sutures eliminate the need for knot tying, providing uniform tension distribution along the pexy line and contributing to time savings without increasing postoperative complications.

Among these, absorbable fixation straps (AFSs) offer an effective alternative by allowing secure gastric fixation without the need for intracorporeal sutures. Previous cadaveric [12] and clinical [7,13] studies have evaluated the feasibility of using AFSs in sutureless total laparoscopic gastropexy in dogs. This three-port technique employs the Ethicon Securestrap™ Absorbable Strap Fixation Device, which deploys straps made from polydioxanone and a copolymer of lactide and glycolide. These studies demonstrated that an AFS enables the creation of strong and lasting adhesions while significantly reducing operative time and simplifying the procedure.

Given the elective nature of prophylactic gastropexy, there is a growing interest in combining laparoscopic gastropexy with ovariectomy into a single minimally invasive surgical session [5,6,14]. This approach offers the potential to further reduce overall surgical trauma, anesthesia exposure, and recovery time while maximizing the benefits of both interventions [14].

The aim of this study to report the first clinical application of a combined protocol integrating laparoscopic sutureless gastropexy with absorbable fixation straps (TLG-SS) and laparoscopic ovariectomy (LOVE) using a standardized three-port minimally invasive approach.

## 2. Materials and Methods

### 2.1. Animal Welfare and Ethics Statement

This study was a prospective case series. Dogs involved in the study are owned by clients, and their recruitment was authorized by owners through written consent. The study was approved by the Ethics Committee for Veterinary Clinical and Zootechnical Studies of the Department of Precision and Regenerative Medicine and Jonian Area. (Nr. 2873-2 III/13, 30 April 2025).

### 2.2. Population

#### 2.2.1. Inclusion Criteria

Female dogs of breeds predisposed to gastric dilatation-volvulus (GDV) were included in the study, provided that the owners had previously expressed the intention to have their dogs sterilized.

Exclusion criteria included the presence of concurrent disorders, reproductive tract disease, anesthetic risk factors, abnormalities detected in preoperative blood panel, or lack of owner compliance with the scheduled follow-up visits.

#### 2.2.2. Surgical Procedure

All dogs were premedicated with intramuscular (IM) administration of 10 μgkg^−1^ of acepromazine (Prequillan; Fatro, Italy; 10 mg mL^–1^) and, after 15 min, 0.3 mgkg^−1^ of methadone (Semfortan; Dechra, Italy; 10 mg mL^–1^). When adequate sedation was achieved, a cephalic vein was cannulated for the intravenous (IV) administration of fluids and drugs. General anaesthesia was induced with propofol (Fresenius Kabi Propofol 10 mg mL^−1^) at 5 mLkg^−1^ IV, and the animals were intubated and connected to a re-breathing circuit. Anaesthesia was maintained with inhaled isoflurane and pure oxygen (FiO2 1). All dogs were mechanically ventilated during the entire procedure in volume-controlled mode (Servo-I; Maquet, Germany) with a tidal volume of 15 mLkg^−1^, inspiratory to an expiratory ratio of 1:2, inspiratory pause of 25% of the inspiratory time, and PEEP of 5 cmH_2_O. The respiratory rate (RR) was adjusted for the end-tidal carbon dioxide level (Pe′CO_2_), which was maintained between 40 and 55 mmHg.

Patients were placed in dorsal recumbency on a special reclining table. The abdominal skin was prepared aseptically, and the surgical field was delimited by sterile surgical drapes. All dogs underwent a total laparoscopic 3-port approach: a central port was placed caudally at the umbilicus. The choice of central port size varied according to weight and BCS: a wound retractor with laparoscopic cannula cap was used in bitches weighing more than 40 kg and/or with a BCS greater than 3, whereas a 12 mm cannula was used in all other cases. A 5 mm cranial port was placed caudally at the left third mammary gland, and a 5 mm caudal port was placed caudally at the right fourth mammary gland.

**Figure 1:**
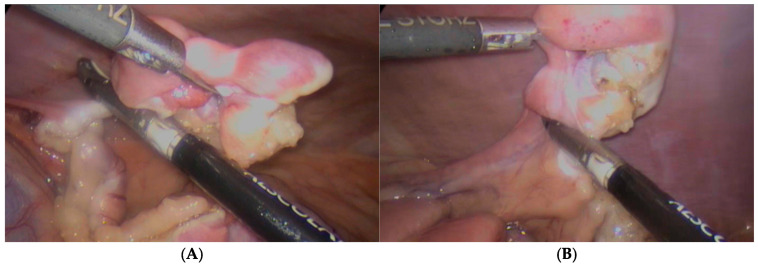
Surgical views during laparoscopic ovariectomy. (**A**) Left ovary: patient tilted 45° to the right; (**B**) Right ovary: patient tilted 45° to the left. The image sequence demonstrates how the proposed three-port configuration provides optimal access and instrument mobility for both ovaries, confirming the adequacy of this setup in the combined procedure.

After port placement and induction of pneumoperitoneum to a maximum intrabdominal pressure of 8 mmHg, LOVE was performed as first phase. In the first step, the surgeon focused on the left ovary: the patient was 45° tilted on the right side, the ovary was grasped directly with laparoscopic forceps and coagulation and resection of the mesovarium, and ovarian pedicle was performed with an advances bipolar vessel sealing device (Caiman, BBRAUN, Milan, Italy). The ovary was grasped with forceps and extracted through the central port. Next, the patient was tilted to the left side and the other ovary was removed using the same technique. Figure 1.

**Figure 2:**
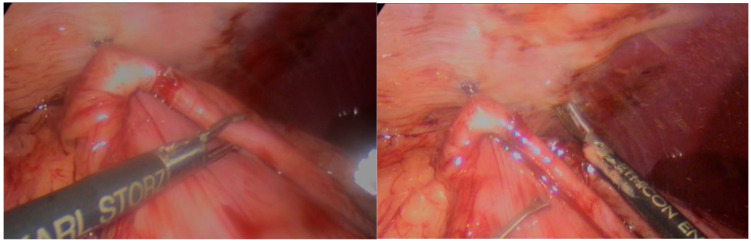
Application of an AFS to suspend the stomach to the abdominal wall. Scarification of abdominal wall seromuscular with a harmonic scalpel.

Finally, with the animal still rotated on the left flank with an angle of 20°, TLG-SS was performed on the right side of the abdomen, as described elsewhere [7,13]. Briefly, the pyloric antrum was grasped and approached to the right abdominal wall at the 12th rib, then two AFSs were applied to fix the stomach to the abdominal wall (one cranial and one caudal); Figure 2.

**Figure 3:**
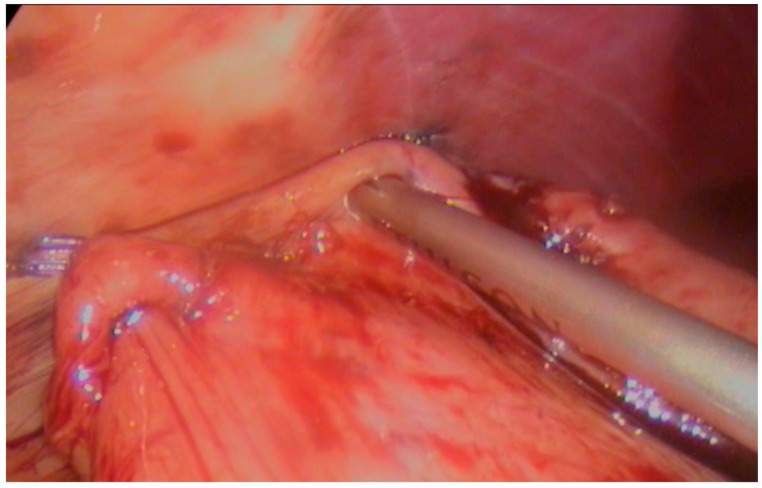
Application of a proper number of AFSs to cover the cauterization line and complete stomach fixation.

Scarification of seromuscular of the stomach and abdominal wall serosa were then performed with a harmonic scalpel (Ultracision, J&J, Cincinnati, OH, USA). Finally, the gastropexy was completed by applying an appropriate number of AFSs to cover the cautery line; Figure 3.

At the end of the two procedures, the pneumoperitoneum was interrupted, the cannulas were removed, and the operative incisions were routinely sutured.

#### 2.2.3. Postoperative Regimen

The postoperative regimen included an analgesic plan (tramadol 4 mg/kg BID, PO for 3 days) combined with anti-inflammatory (Robenacoxib 1 mg/kg SIID, PO for 3 days) and antibiotic (Amoxiclavulanic acid 20 mg/kg BID, PO for 3 days) therapy. Sutures were removed at a mean of 10 days after surgery.

#### 2.2.4. Surgical Variables and Complications Considered

The time required for port placement (in minutes) was recorded at the beginning of each procedure. Total operative time (in minutes) was measured as skin-to-skin time, defined as the interval from the initial skin incision for port insertion to final port removal and skin closure. This value encompassed the duration of both LOVE and TLG-SS.

Ovariectomy time was defined as the interval between the initiation of resection of the first ovary and the extraction of the second ovary. Gastropexy time was measured from the placement of the first AFS to the completion of the pexy.

Intraoperative complications were documented and classified as either minor or major. Minor complications were those that did not require significant deviation from the standard surgical protocol and resolved spontaneously or with minimal intervention. Examples include minor bleeding, difficulty in instrument manipulation, suboptimal visualization, or intraoperative rupture of the AFS. Major complications were defined as events necessitating substantial changes to the surgical plan, such as emergency conversion to open surgery. These included events such as pneumothorax, subcutaneous emphysema at the port sites, or splenic laceration.

Intraoperative bleeding was subjectively graded as mild, moderate, or severe. Mild bleeding was defined as self-limiting, moderate bleeding required the use of electrosurgical devices for hemostasis, and severe bleeding was deemed uncontrollable through laparoscopic means.

In cases of major intraoperative complications—including failed gastropexy, significant visceral injury, or uncontrolled hemorrhage—the procedure was converted to open surgery (celiotomy), with completion of ovariectomy and/or gastropexy via a conventional approach on an emergency basis.

#### 2.2.5. Follow-Up

Follow-up was performed 7 days after surgery, and again 14 days after surgery to evaluate complications and surgical wound conditions.

Postoperative complications were classified as major and minor. Minor postoperative complications were defined as self-limited or resolved with supportive care. Examples of minor complications include bruising, swelling, erythema, or subcutaneous emphysema near the incision site. Major postoperative complications were defined as those requiring postoperative veterinary intervention. For example, surgical site infection or persistent seroma were considered major complications. At the 14-day follow-up, owners were asked to complete a simplified questionnaire to provide an overall evaluation of their dog’s postoperative management. Satisfaction was rated on a five-point scale ranging from ‘not at all satisfied,’ ‘slightly satisfied,’ ‘moderately satisfied,’ ‘very satisfied,’ to ‘excellently satisfied.’

## 3. Results

### 3.1. Population Study

Six dogs were recruited, specifically three Great Danes, two German shepherds, and one Belgian Malinois.

The mean weight of the subjects was 41.7 kg ± 4.97 (range: 35–47 kg), the mean age was 6.3 years ± 0.8 (range: 5–7 years), and the mean BCS was 3/5 ± 0.4 (range: 3–4).

### 3.2. Surgical Approach

All subjects underwent right flank gastropexy with TLG-SS technique, and the number of absorbable fixation straps placed was 8–9 in order to cover the scarification line.

The mean time for ports placement was 6.50 min ± 1.05 (range: 5–8 min). The mean recorded time for skin-to-skin surgery was 29.0 min ± 3.52 (range: 26–35 min). The mean time for ovariectomy was 7.50 min ± 1.38 (range: 6–10 min). The mean time for gastropexy was 9.33 min ± 2.58 (range: 7–14 min).

### 3.3. Intraoperative Complications

During gastropexy, in one subject it was necessary to cross the falciform ligament with the surgical instrument to facilitate surgical maneuvering, but no moderate or major complications were encountered. The surgical technique used for ovariectomy and gastropexy was successfully performed in all the subjects with no major complications and only mild self-limited bleeding. There was no need to convert the procedure to open surgery in any case.

### 3.4. Postoperative Management

In no case was additional analgesic treatment required. The patients were all discharged within a few hours of performing surgery.

### 3.5. Follow-Up and Postoperative Complications

Clinical follow-up at 7 and 14 days revealed only mild left cranial portal reactivity in one dog, which required no further intervention.

No dogs showed gastrointestinal symptoms, and the level of owner satisfaction was rated as excellent.

**Figure 4:**
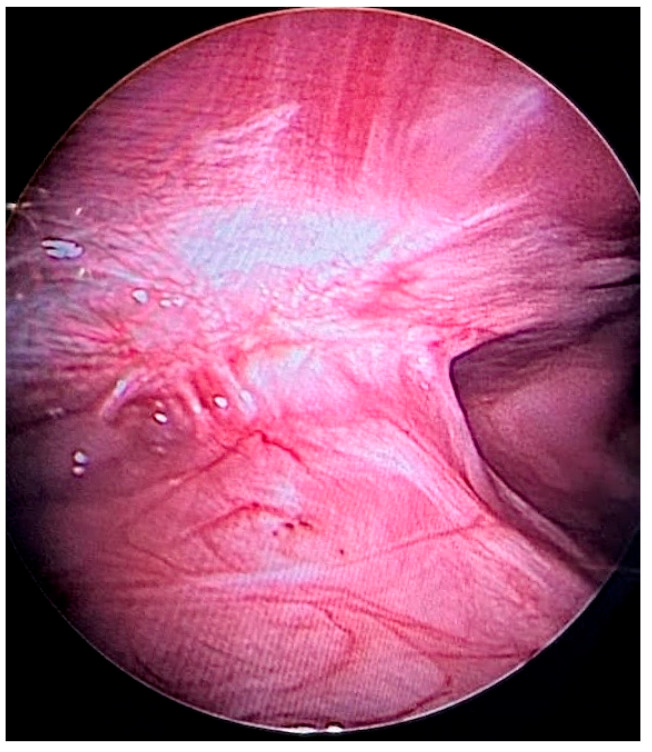
Intraoperative visualization of the gastropexy site three months after surgery. At follow-up laparoscopic evaluation, the gastropexy appeared in situ, stable, and well-integrated, with no signs of displacement or dehiscence. The absorbable fixation staples (AFSs) were no longer visible, suggesting resorption and tissue integration at the fixation site (courtesy of Dr. Abbondanza S., Rimini, Italy).

In one case, there was the opportunity to visualize the gastropexy three months after surgery because the dog underwent another laparoscopic procedure unrelated to this study (liver biopsy), performed at a different hospital. The gastropexy was in situ and stable three months after surgery, and the AFSs had been not visible and presumably reabsorbed; Figure 4.

## 4. Discussion

The study evaluated the feasibility and efficacy of performing laparoscopic ovariectomy and TLG-SS with AFS for prophylactic purposes in GDV susceptible bitches in a single surgical session.

The surgical technique used for LOVE and TLG-ss was successfully performed in all six subjects without major complications and without the need to change port placement or convert the procedure to traditional open surgery. In the three-port approach for LOVE previously described in the literature, one trocar is placed caudally to the umbilicus and the other two are placed on the *linea alba* cranially and caudally to the first one [15,16]. In this study a three-port approach was applied to perform both TLG-SS and LOVE, based on the setting described by Lacitignola et al. [7,13], involving the use of three ports: one at the level of the left cranial abdomen lateral to the rectus abdominis and caudal to the third mammary gland, one caudal to the umbilicus and one at the level of the right caudal abdomen lateral to the rectus abdominis, and one caudal to the fourth mammary gland. The placement of the three ports allowed optimal visualization and easy access to the abdominal organs, facilitating the procedure and minimizing the risk of complications. The ports used allowed the two procedures to be performed without changing the portal placement of each procedure.

A ring wound retractor with a laparoscopic cap was used as the central (umbilical) port in four subjects weighing more than 40 kg, and a 12 mm cannula was used in the other cases (*n* = 2).

The use of a ring wound retractor in dogs over 40 kg improved access by minimizing complications due to accidental perforation of organs during port placement. In addition, as reported in the literature, maintaining a 2.5 cm surgical incision facilitates ovarian extraction after resection, minimizes the risk of specimen loss during extraction, and allows pneumoperitoneum to be easily restored without the risk of gas leakage [17,18,19,20,21,22].

In subjects with a 12 mm central port, ovarian extraction was more difficult; however, there was no need to increase the size of the port.

When performing a combined ovariectomy and gastropexy in bitches, it is recommended to begin with removal of the left ovary for several practical and anatomical reasons that simplify the procedure and improve its overall effectiveness [5,6,14]. First, the gastropexy requires the stomach to be attached to the abdominal wall, and this process requires adequate space and visibility. Starting from the left ovary provides more space on the right side of the abdomen where gastropexy is performed. This greatly facilitates access and manipulation of the stomach, making the procedure easier.

From a surgical perspective, following this sequence establishes a logical and continuous flow of the procedure. This systematic approach can reduce the risk of complications and ensure a smoother and safer procedure without having to change the patient’s position too often.

The number of AFSs applied was 8–9 and was sufficient to ensure the creation of a good gastropexy, as shown in previous studies [7,12,21].

During the gastropexy in one subject, it was necessary to cross the falciform ligament with the surgical instrument to facilitate surgical maneuvering, but no moderate or severe complications were encountered.

The surgical technique used for ovariectomy and gastropexy was successfully performed in all subjects, with no major complications and only mild self-limited bleeding. In no case was conversion to open surgery required, supporting the technical feasibility and favorable short-term tolerance of the laparoscopic protocol.

These results are consistent with those reported by other authors who have associated LOVE with TLG with barbed sutures or LAG [8,9,10,11,23].

No additional analgesic treatment was required in any case. All patients were discharged within a few hours of surgery. These results are consistent with other minimally invasive techniques [3,15].

In this study, clinical follow-up at 7 and 14 days revealed only mild left cranial portal reactivity in one subject, which was self-limited and did not require further intervention. No dogs exhibited gastrointestinal symptoms and owner satisfaction was rated as excellent.

The incidence of postoperative complications was comparable to that reported for TLG-SS with AFSs alone [7,13] and for other totally laparoscopic techniques [2,10,11,23,24], where postoperative bruising, swelling, or erythema resolved within 3–5 days. This is in marked contrast to laparoscopic-assisted [25] or open techniques [26,27] where the incidence of postoperative seroma or complications requiring veterinary intervention is much higher.

The average operative time for ovariectomy (including both ovaries) was 7.50 min (±1.38), while gastropexy required an average of 9.33 min (±2.58). The total skin-to-skin surgical time was 29 min (±3.52). These results demonstrate a high degree of procedural efficiency and smooth intraoperative execution. When compared to previously reported durations for combined laparoscopic ovariectomy and laparoscopic-assisted gastropexy—ranging from 39 to 77.4 min—our findings suggest a substantially shorter surgical time [6,14,19,28].

Notably, Leonardi et al. reported a mean duration of 48 min for the combination of laparoscopic ovariectomy and total laparoscopic gastropexy using barbed sutures [5], which remains significantly longer than the operative times achieved in the present study. This marked reduction in surgical time may be attributed to a simplified and standardized technique, and it contributes not only to minimizing the duration of anesthesia but also to enhancing procedural throughput and overall surgical productivity.

The present findings are consistent with previous studies conducted by our group, which demonstrated that this technique is associated with a significantly shorter learning curve compared to other gastropexy approaches [7,13]. In particular, operative time was reduced from 19 to 7 min after only six procedures, whereas similar reductions in procedural time for alternative techniques typically require between 12 [28] and 30 procedures [8,11,23].

These preliminary data highlight the procedural simplicity of the method, which may prove advantageous in clinical settings. However, its reproducibility and applicability across varying surgical experience levels remain to be validated through larger studies. This design rationale supports its integration within elective surgical protocols and may facilitate its adoption by surgeons with limited experience in minimally invasive surgery. Ultimately, the reduced learning curve contributes to improved surgical efficiency and promotes broader dissemination of the technique in veterinary clinical practice.

In one case, there was the opportunity to visualize the gastropexy three months after surgery because the subject underwent another laparoscopic procedure unrelated to this study. Three months following surgery, the gastropexy was excellent and stable, and the AFSs were no longer visible, implying that they were essentially completely reabsorbed. This finding is in line with post-mortem evaluation made by Lacitignola et al. 20 months after surgery, which showed the creation of an adhesion band between peritoneal and stomach serosa with no residual AFSs [7].

This study presents several limitations that must be acknowledged. First and foremost, the small sample size (*n* = 6) and the absence of a control group inherently limit the ability to draw statistical comparisons. Despite the use of strict inclusion and exclusion criteria, the descriptive nature of the study precludes definitive conclusions on clinical efficacy.

Secondly, no standardized pain scoring system was employed to objectively assess postoperative discomfort, nor were imaging modalities such as ultrasonography or endoscopy used to evaluate gastropexy integrity over time. While these limitations restrict the interpretation of long-term effectiveness and recurrence prevention, it is important to clarify that such outcome metrics were not the aim of the present investigation. The TLG-SS technique—including its biomechanical safety, risk of mucosal penetration, port placement optimization, complication profile, ultrasonographic follow-up, and even histological confirmation—has already been extensively investigated and validated in previous publications by our group [7,12,13].

For this reason, the present study focused exclusively on the technical feasibility and perioperative management of the combined laparoscopic approach (TLG-SS + LOVE), and postoperative assessment was limited to clinical parameters and short-term follow-up. The lack of a validated survey instrument for owner satisfaction is also acknowledged and has been clarified in the Methods section. In future studies, integration of validated pain scoring systems (e.g., Glasgow Composite Measure Pain Scale), imaging follow-up, and longer-term monitoring will be essential to fully characterize the benefits and durability of this combined technique.

Finally, we recognize that the degree of technical innovation in the present study is limited, as both LOVE and TLG-SS are well-established procedures. However, to our knowledge, this is the first clinical report to describe their combined application using a fully laparoscopic three-port protocol with absorbable fixation straps. We considered it clinically relevant to document this integration, given the growing adoption of both techniques individually and the absence of published data on their standardized combination.

## 5. Conclusions

This prospective case series suggest that the combination of total laparoscopic sutureless gastropexy (TLG-SS) using absorbable fixation straps (AFSs) and laparoscopic ovariectomy (LOVE) in dogs predisposed to GDV is technically feasible and well-tolerated in the short term when performed in a single minimally invasive surgical session. The three-port approach proved technically efficient, providing optimal visualization and access to abdominal organs without the need for port repositioning. All procedures were completed without major intraoperative or postoperative complications, and no conversions to open surgery were required. Surgical times were markedly reduced compared to previously reported combined techniques, with a mean skin-to-skin time of 29 min, thus minimizing anesthesia duration and enhancing perioperative efficiency. Postoperative recovery was uneventful in all cases, with minimal discomfort, no need for additional analgesia, and high owner satisfaction. Furthermore, the technique demonstrated a favorable learning curve, suggesting that it may be a viable and accessible option even for surgeons with limited experience in advanced laparoscopy. These findings support the potential use of this dual minimally invasive protocol in elective surgical settings, although further controlled studies are required to confirm its efficacy, safety, and long-term reliability.

## Data Availability

All data were included in the manuscript.

## Abbreviations

The following abbreviations are used in this manuscript:

GDV	Gastric Dilatation and Volvulus
LAG	Laparoscopic Assisted Gastropexy
TLG	Total Laparoscopic Gastropexy
TLG-SS	Total Laparoscopic Gastropexy-Secure Strap
AFS	Absorbable Fixation Strap
LOVE	Laparoscopic Ovariectomy
